# Age-related increase of VGF-expression in T lymphocytes

**DOI:** 10.18632/aging.100656

**Published:** 2014-05-04

**Authors:** Stefan Busse, Johann Steiner, Justus Micheel, Henrik Dobrowolny, Christian Mawrin, Tim J. Krause, Michael Adamaszek, Bernhard Bogerts, Ursula Bommhardt, Roland Hartig, Mandy Busse

**Affiliations:** ^1^ Department of Psychiatry, University of Magdeburg, Magdeburg, Germany; ^2^ Center for Behavioral Brain Sciences, Magdeburg, Germany; ^3^ Pembroke College, University of Cambridge, Cambridge, UK; ^4^ Department of Neuropathology, University of Magdeburg, Magdeburg, Germany; ^5^ Center of Neurological Rehabilitation, University of Leipzig, Leipzig, Germany; ^6^ Institute of Immunology, University of Magdeburg, Magdeburg, Germany; ^7^ Department of Pediatric Pulmonology, Allergology & Neonatology, Medical University of Hannover, Hannover, Germany

**Keywords:** aging, CD3 lymphocytes, VGF expression, Rapamycin

## Abstract

VGF is a protein expressed by neurons and processed into several peptides. It plays a role in energy homeostasis and promotes growth and survival. Recently, VGF mRNA was detected in peripheral leukocytes. Since it is known that aging is associated with a decrease in the development and function of neuronal as well as immune cells, we addressed the question whether a peripheral expression of VGF by CD3+ T cells and CD56+ NK cells is correlated with age. Therefore, the frequency of VGF+CD3+ and VGF+CD56+ cells was determined in mentally healthy volunteers aged between 22 and 88. We found an age-dependent increase in the number of VGF+CD3+ T cells that correlated with HbA1c and the body mass index (BMI). VGF-expression by NK cells was age-independent. Blockade of VGF reduced proliferation and secretion of cytokines such as IL-2, IL-17A, IL-1β, IL-10 and TNF by CD3+ T cells and PBMCs. Rapamycin-mediated T cell blockade significantly reduced the frequency of VGF-expressing T cells. We conclude that VGF contributes to survival and function of peripheral T cells. The age-dependent increase in VGF-expression could serve as mechanism that counterregulates the decrease in functionality of T lymphocytes.

## INTRODUCTION

Aging is becoming of increasing importance, because the cohort of older adults constitutes the fastest growing population in the world for the first time in history. It has been predicted that about by the year 2025 the population older than 65 years will be increasing 3.5 times as rapidly as the total population [1]. Therefore, studies of the age-dependent molecular and cellular mechanisms are of rising interest. Aging is associated with a decline in the function of the immune system, characterized by dramatic changes on the cellular and

systemic level that affect the innate as well as the adaptive immune system: The innate immune system, represented e.g. by natural killer (NK) cells, macro-phages and dendritic cells (DCs), shows an age-associated impaired function to trigger T-cell responses [2]. The output of naive T cells, part of the adaptive immunity, declines with age as a result of the thymic involution [3]. However, elderly people have significantly higher number of T cells that are specific for persistent viruses, such as EBV and CMV [4]. This so-called immunosenescence is further characterized by an inverted CD4+/CD8+ T cell ratio, a shift from Th1 to Th2 cell induction following T cell activation that influences the balance between humoral and cell-mediated immune responses. This altered cytokine profile is probably the result of an increased ratio of memory to naive T cells [5, 6]. The cytokine milieu is modulated during aging also in another direction: elevated levels of circulating proinflammatory cytokines, like IL-6 and TNFα [7] were detected in elderly people. It is known that chronic inflammation has a negative impact on tissues, organs, and health in general. Increased levels of IL-6 and TNFα and their receptors are significant independent risk factors for mortality and morbidity in the aged population [8]. As a consequence of immunosenescence, there is an increased incidence of age-related pathologies [9-12]: Senescent T cells may play a role of cardiovascular disease [13], atherosclerosis [14], myocardial infarction [15] and type 2 diabetes mellitus (T2DM) [16]. There is abundant communication between the central nervous system (CNS) and the immune system. For instance, T cells are important in functions of the healthy brain such as spatial learning and memory [17, 18] and adult neurogenesis [19]. Senescent T cells are also involved in neurodegenerative disorders such as Alzheimer's disease [20, 21] or vascular dementia [22]. Therefore, new therapeutic treatment options for neurodegenerative diseases are needed, such as Rapamycin. Rapamycin, a macrolide immuno-suppressant used to prevent rejections following organ transplantation via inhibition of T and B cell proliferation, was shown to reduce amyloid-beta levels, to abolish cognitive deficits in mouse models of Alzheimer's disease [23] and to suppress brain aging in rats [24].

www.impactaging.com 441 AGING, June 2014, Vol. 6 No.6VGF is a neuronal polypeptide first identified as a cDNA clone from plate V of the nerve growth factor (NGF)-induced rat pheochromocytoma (PC12) cell cDNA library [25]. The VGF protein is widely expressed in the neurons in brain and hippocampal synaptic activity [26] and is subsequently processed into more than 10 different peptides; these peptides play an important role by improving plasticity, neurogenesis, energy homeostasis, pain modulation and sexual behaviour (for a recent review, see [27]). VGF is abundantly expressed in the thalamus and promotes dendritic growth and survival of cortical neurons [28]. Certain VGF peptides are also involved in the regulation of food uptake and energy balance. VGF- deficient mice are smaller and thinner than wild type mice [29]. However, some VGF peptides like TLQP-21 have an opposite effect on fat deposition and trigger lipolysis or induce resistance to obesity by reducing fat accumulation [30].

After enzymatic processing, smaller VGF-derived peptides are secreted into the cerebrospinal fluid (CSF) or blood. Therefore, some VGF fragments were considered as biomarkers for neurological and psychiatric disorders, such as Alzheimer's disease, frontotemporal dementia and schizophrenia [31].

Recently, Cattaneo et al. detected VGF mRNA expression also in human leukocytes [32]. Since it was shown that peripheral T cells express several neurotransmitter receptors, proteins and metabolites originally identified in cells of the nervous system [33], we wondered if T cells express VGF as well. Moreover, we questioned if blocking of VGF has any functional consequences and whether VGF levels are age-dependent. Therefore, we investigated the VGF expression in T cells of mentally healthy persons aged between 22 and 88 years using flow cytometry.

Obesity is the hallmark of the metabolic syndrome that represents a cluster of different disorders which predispose people for the development of chronic metabolic diseases including T2DM and cardiovascular diseases [34]. As a consequence of a sedentary lifestyle and increased energy uptake, an enlargement of adipose tissue takes place that is associated with an inflammatory immune response which leads to adipose tissue dysfunction [35]. The number of CD4+ and CD8+ T cells are elevated in obese human adipose tissue, which indicates changes in T-cell infiltration in human adipose tissue [36]. Therefore, the T-cell compartment balance is important for the regulation of adipose tissue homeostasis. Since VGF potentially influences energy homeostasis, we evaluated the association between HbA1c and the body mass index (BMI) and the VGF expression in T cells. Furthermore, we determined the frequency of VGF-expressing CD56+ NK cells during senescence.

We detected an increase of VGF-expressing CD3+ T cells during aging. This increase was correlated with the level of HbA1c and the BMI. *In vitro* blockade of VGF using an antibody reduced proliferation and the cytokine production by CD3+ T cells and peripheral blood mononuclear cells (PBMCs). However, we detected no changes in the expression of VGF by NK cells with increasing age.

Our data suggest that VGF could contribute to survival and function of peripheral T cells. We speculate that the age-dependent increase in VGF-expression could serve as a mechanism that counterregulates the decrease in functionality of T cells during senescence.

## RESULTS

### Study cohort

42 healthy gender-matched volunteers (23 females, age 22-88 years; 19 males; 22-86 years) were included in the study. The demographic features of the mentally healthy volunteers are summarized in suppl. table 1. Routine blood parameters including lipid status of all

volunteers were analyzed and no changes were found indicating a severe illness. Most of the younger volunteers had no underlying disease and used no medication, while some of the older suffered from arterial hypertension, hyperlipidemia, hypothyroidism, chronic obstructive pulmonary disease, cardiac insufficiency, hyperuricemia or osteoporosis and had to be under medication. Mini mental status-test (MMST) of all volunteers was 29 or 30 (suppl. table 1).

### Age-dependent expression of VGF by CD3+ T cells and CD56+ NK cells

**Figure 1:**
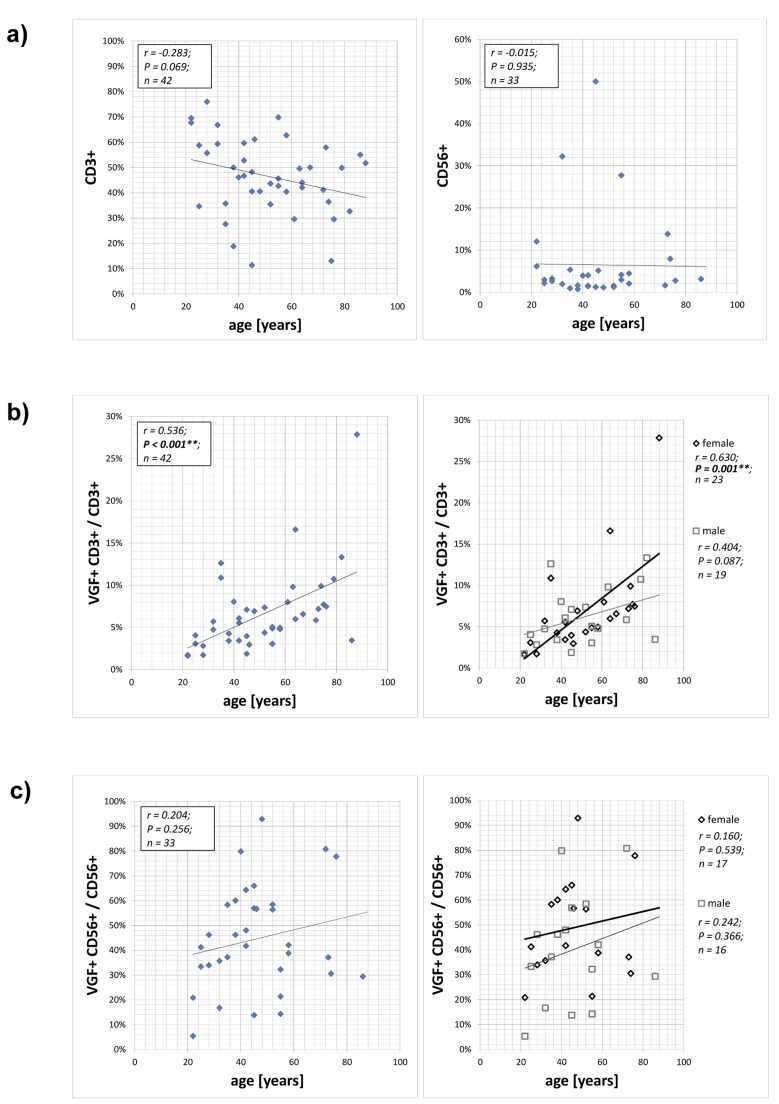
Expression of VGF on CD3+ T cells and CD56+ NK cells PBMCs isolated from 42 healthy volunteers aged between 22 and 88 years without psychiatric diagnosis were stained with anti-VGF (D20) and anti-CD3 or anti-CD56 and were analyzed using flow cytometry. The numbers of CD3+ T cells and CD56+ NK cells depending on the age of the persons is shown in (**a**). The frequency of VGF-expressing CD3+ cells within the T lymphocytes population is correlated with the age of the cohort (**b**; left) and further subdivided according to the gender (**b**; right). The proportion of VGF-expressing CD56+ cells within the NK cell population is shown in dependence with the age of the cohort (**c**; left) and further subdivided according to the gender (**c**; right).

A flow cytometry approach was used to determine the expression of VGF-positive CD3+ T cells (VGF+CD3+; an example is shown in suppl. fig. 1) and CD56+ NK cells (VGF+CD56+). The number of CD3+ T cells tended to decrease with age (p=0.069; Figure 1a). The frequency of VGF-expressing CD3+ T lymphocytes among the CD3+ lymphocytes (VGF+CD3+/ CD3+) ranged between 1.62% and 27.85% and showed an age-dependent increase (p=0.001; Figure 1b; suppl. table 2 and suppl. fig. 2). This increase was more pronounced in female (p=0.001) than in male volunteers (p=0.087). The number of CD56+ NK cells was not age-dependent (p=0.423; Figure 1a). The percentage of VGF-expressing CD56+ NK cells in the CD56+ NK cell population (VGF+CD56+/ CD56+) ranged between 5.30% and 80.77%, but this increase did not correlate with age, neither in the female nor in the male cohort (Figure 1c; suppl. table 2 and suppl. fig. 2).

**Figure 2:**
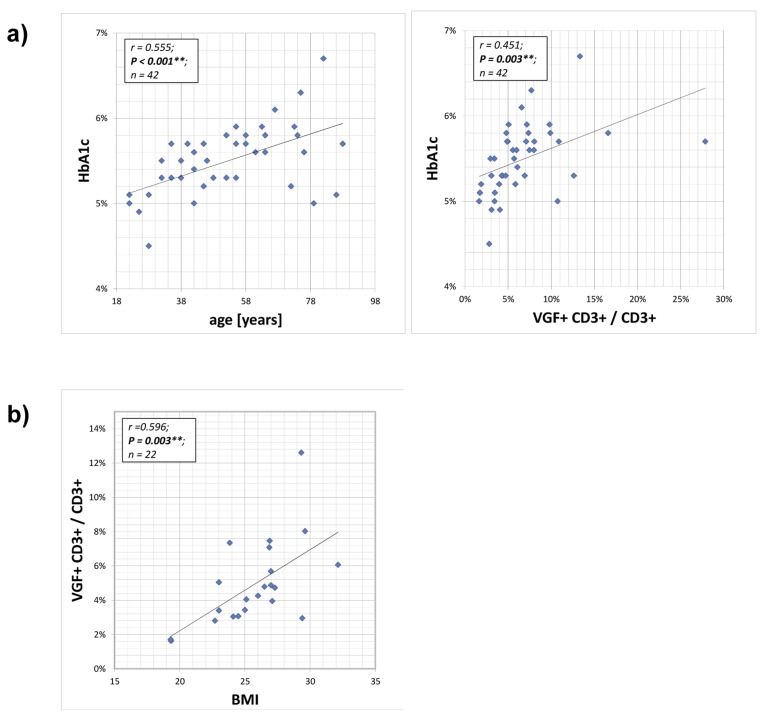
Correlation between HB1Ac and VGF-expressing CD3+ T cells The HbA1c (glycated haemoglobin) level provides an integrated average blood glucose measure and was determined during routine blood analysis. The HbA1c value of the cohort increased with age and was positively correlated with frequency of VGF-expressing CD3+ cells within the T cell population (**a**). Also the body mass index (BMI) increased with a rising number of VGF+CD3+ T cells (**b**).

### Correlation between HB1Ac, BMI and VGF-expressing CD3+ T cells

Glycated haemoglobin (HbA1c) levels provide an 8–12 week integrated average blood glucose measure and were determined during routine blood analysis. The HbA1c values of the volunteers increased statistically with age (p=0.001). Therefore, a positive correlation between HbA1c and VGF+CD3+/ CD3+ T lymphocytes (p=0.003) and the body mass index (BMI; not from all volunteers available; p=0.003) and the frequency of VGF+CD3+/ CD3+ T cells was calculated. For regression analysis, we used VGF+CD3+/ CD3+ as dependent factor and HB1Ac, BMI and age as independent variables. When considering all three confounders, age is the significant and relevant factor.

### VGF induces proliferation and cytokine production by T cells

**Figure 3:**
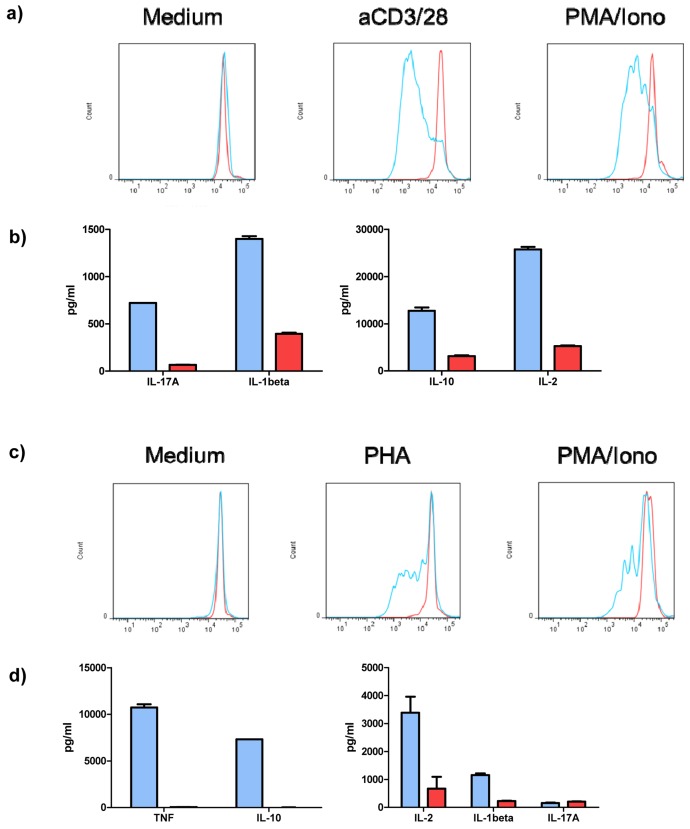
VGF induces proliferation and cytokine production by T cells and PBMCs Isolated T cells were stimulated with aCD3/28 or PMA/Ionomycin (as positive control) or were left untreated (medium control) for 3 days in the presence of anti-VGF (D20; red) or an isotype-matched control Ab (blue). Division of cells is detected by loss of fluorescence intensity of cells labeled with CFSE. Blockade of VGF inhibited the proliferation of T cells stimulated with aCD3/28 or PMA/Ionomycin (**a**) and reduced the production of cytokines in T cell cultures stimulated with aCD3/28 (**b**). Blocking of VGF diminished the division of PBMCs stimulated with PHA or PMA/Ionomycin (as positive control; **c**) and also the secretion of cytokines in PHA-stimulated PBMC cultures.

We addressed the question whether the inhibition of VGF has any functional consequences for CD3+ T cells. Therefore, isolated CD3+ T cells were stimulated with Dynabeads Human T-Activator CD3/CD28 (and PMA/Ionomycin as positive control) in the presence of either anti-VGF Ab or an isotype-matched control for three days and then the cytokine levels in the supernatant were measured. To determine proliferation, CD3+ T cells were additionally labeled with CFSE before culturing (Figure 3a). The treatment of T cells with anti-VGF during stimulation with aCD3/CD28 diminished the production of the T cell survival factor IL-2, but also of the Th17-mediated cytokine IL-17A, the Th1-associated cytokine IL-1β and the Treg/ Th2-cytokine IL-10 (Figure 3b). Blockade of VGF also strongly inhibited the proliferation of CD3+ T cells.

### VGF induces proliferation and cytokine production in PBMCs

Stimulation of CD3+ T cells with aCD3/CD28 acts independent of the presence of antigen-presenting cells (APCs). Whether VGF also influenced the interaction between T cells and APCs was determined by stimulation of PBMCs with PHA (and PMA/Ionomycin as positive control) in the presence of either anti-VGF Ab or an isotype-matched control. CFSE-labeled PBMCs were used to determine proliferation (Figure 3c).

The treatment of PHA-stimulated PBMCs with anti-VGF Ab inhibited the secretion of TNF-α and IL-10 and decreased the production of IL-2 and IL-1β. However, the level of IL-17A, which is also produced by other cell types such as macrophages or monocytes, was unchanged (Figure 3d). Blockage of VGF severely diminished the proliferation of the PBMCs induced by PHA.

### VGF expression is reduced upon rapamycin treatment

**Figure 4:**
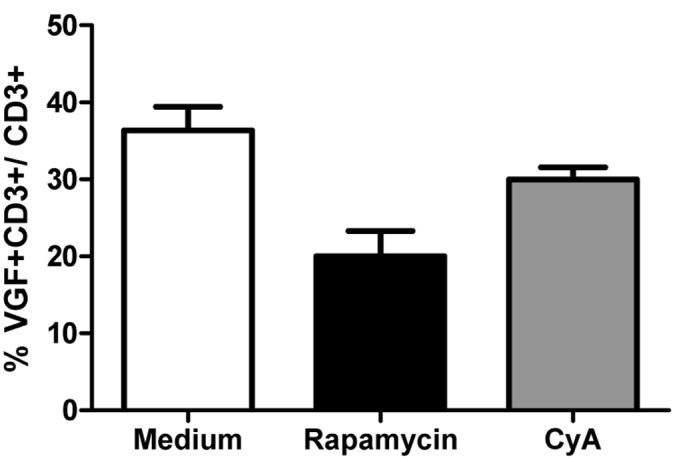
Rapamycin-treated T cells down-regulate VGF Isolated PBMCs were cultured in medium in the presence or absence of either Rapamycin or Cyclosporine A for 24h. Afterwards, the frequency of VGF-expressing CD3+ T cells was determined using flow cytometry (*p<0.05; one-way ANOVA).

Rapamycin and cyclosporine A are both immunosuppressive drugs which inhibit the activation of T cells and therefore are used to prevent organ rejections. To determine their function in the expression of VGF by T cells, PBMC were incubated in medium with or without rapamycin or cyclosporine A. Treatment with rapamycin reduced the expression of VGF (p=0.0154, Figure 4) compared to medium. Also cyclosporine A was able to reduce the frequency of VGF+CD3+ T cells, however only to a smaller extent.

## DISCUSSION

To our knowledge this is the first report showing that the neuronal polypeptide VGF is expressed by peripheral T cells and, to a smaller extent, by NK cells. The expression of VGF by T lymphocytes increased with age.

It was shown that T cells protect neurons from degeneration and this neuroprotective effect is mediated via the production of neurotrophins [37, 38], the modulation of glutamate release by astrocytes and microglia [39, 40], the regulation of innate immunity at the site of injury [41]. Other still unexplored mechanisms may be involved. Since it was described that immune cells express a broad range of receptors specific for adrenaline, noradrenaline and dopamine it was also shown that these neurotransmitters are involved in both the propagation and resolution of immune responses, splenic T cells are able to produce acetylcholine [42]. T cells can also secrete dopamine by themselves. Dopamine signalling through D1 and D5 receptors in Treg cells attenuated their suppressive properties [43]. Moreover, pro-cognitive functions of T cells were discussed as well (reviewed in [44]). Furthermore, neurotransmitters and neuropeptides can modulate the functions of immune cells such as T cells, myeloid cells or dendritic cells when released from the CSF into the blood.

Therefore the aim of our study was to explore further hints for an involvement of T cells in the interaction between the CNS and the immune system. Changes in the expression of VGF in the brain or CSF are associated with schizophrenia [45], depressive disorders [46], Parkinson's and Alzheimer's disease [47, 48]. Since Cattaneo et al. [32] showed VGF expression by leukocytes on mRNA level, we used flow cytometry to detect VGF expression on protein level.

Our study showed that blockade of VGF *in vitro* dampened the proliferation and cytokine production by T cells and PBMCs. It is known that senescent T cells have shortened telomeres and increased telomerase activity [49-51] and a dramatically decreased IL-2 production [52]. The important costimulatory molecule CD28 is downregulated while CTLA-4 is upregulated [53-56]. Both molecules bind to CD80 and CD86, and another stimulatory surface molecule, CD27, is absent and this event is coupled with upregulation of the inhibitory molecule CD57. The TCR signal transduction pathways are significantly altered as well [57], resulting in impaired transcription of key effector T cell genes. The result of these events is a decline of T cell immune function in elderly persons such as a blunted immune response to vaccination intended to prevent infections and a reduced ability to fight actual infections. Blocking VGF *in vitro* decreased the secretion of IL-2 indicating that VGF stimulates the production of IL-2. This finding may suggest that VGF has a positive net effect on T cell survival and function.

Moreover, inhibition of T cell activation by rapamycin reduced the expression of VGF by T cells. The importance of TOR, the target of rapamycin, insulin and insulin-like growth factor 1 (IGF-1) signaling pathways for aging was shown by Mikhail V. Blagosklonny, who proposed the hyperfunction theory of aging [58, 59]. According to this new, challenging idea aging is the consequence of continued activity of processes which evolved to optimize development in adulthood, especially excess biosynthesis. TOR regulates biomass within the cell; it inhibits autophagy and promotes protein translation, thereby TOR controls aging. Consequently, intervention in TOR signalling has several anti-aging effects: it increased life span and slowed progression of age-related pathologies in female S6K1 mice [60]. Rapamycin was shown to delay cellular senescence [61, 62], to extent the lifespan in both male and female mice [63] and to suppress brain aging and neurodegeneration in rats [24]. Therefore, Rapamycin could serve as a new therapeutic approach for the treatment of Alzheimer's disease.

TOR is also a key molecular interplayer in nutrient sensing, and calorie restriction is an important aging-delaying intervention. We detected a positive correlation between glycated haemoglobin (HbA1c) and VGF+CD3+/CD3+ T lymphocytes as well between the BMI and the number of VGF-expressing T cells. VGF is involved in maintaining energy balance [64]. Previous studies have shown that VGF knockout mice are thin, small, hypermetabolic and of reduced fat stores [29]. The expression of VGF is not only induced by NGF, BDNF, neurotrophin 3 and fibroblast growth factors, but also by insulin.

CD4+ and CD8+ T cells infiltrate obese human adipose tissue and induce local and systemic inflammation with an increased production of proinflammatory cytokines and provide a major link to the pathogenesis of insulin resistance [65]. Of particular interest are Th17 cells: the percentage of Th17 cells is elevated in blood of T2D patients and was correlated with human BMI, as a measure of adiposity in T2D patients [66]. Moreover, the IL-17 production by T cells correlates also with T2D severity (as measured by HbA1c), further supporting a likely relationship between the Th17 cells and metabolic imbalance. Blocking of VGF in CD3+ T cell cultures stimulated with aCD3/CD28 reduced the level of secreted IL-17 indicating that VGF promotes IL-17 production by T cells. Taken together, our data support Blagosklonny's hyperfunction hypothesis of aging, implicating that T cells are involved in the pathogenesis of insulin resistance and add VGF as a novel biomarker which becomes upregulated by rising HbA1c and the BMI.

Immunosenescence is a complex process that we are just beginning to explore. The age-dependent increase in VGF-expression could serve as a mechanism that counterregulates the decrease in functionality of T lymphocytes to prevent more severe damaging effects during aging.

Compared with T cells, the percentage and also the absolute number of NK cells with a mature phenotype enhances with age [67-69]. No age-dependent changes in the number of VGF-expressing CD56+ NK or total NK cells could be detected. Further investigation will be needed to characterize the nature and function of the VGF-expressing CD3+ T cells in immunosenescence and in age-related pathologies as well.

The study has certain limitations that have to be considered. First, we investigated the expression of VGF in T lymphocytes and NK cells. However, CD3-negative and CD56-negative cells also express VGF. Therefore, a comparative analysis of monocytes and B cells would be important as well. Second, the isolation of T cells by MACS technology resulted in a purity of about 95%. Therefore we do not know how blocking VGF in T cell cultures was influenced by contaminating APCs. Third, we found out that BMI and Hb1Ac influences the expression of VGF in T cells. However, it has to be explored in the future cell culture studies if the supply with nutrients (or starvation) has a direct regulatory effect on the expression of VGF in T cells.

By using the N-terminal antibody (D-20) against VGF, we detected three bands by Western Blot in PBMC and CD3+ T cells, as we had already described for human hypothalamic tissue, which were located at the molecular weights of approximately 90, 60, and 34 kDa [45]. While the 90 kDa protein corresponds to intact VGF, the other bands are proteolytic cleavage products of VGF. However, changes in the presence of the protein/ peptides and the associated meaning for aging and age-related diseases (dementia) are still unknown and currently under intensive investigation.

## METHODS

The study was performed in accordance with German laws, the Declaration of Helsinki, and the guidelines of the local institutional review board (159/11). Written consent was obtained from all healthy persons. We collected 10 ml EDTA blood from 42 healthy persons aged between 22 and 88 years without psychiatric diagnosis. Routine blood analysis (including differential blood cell count, levels of C-reactive protein, glucose, lipids, liver enzymes and thyroid hormones) were performed. None of the persons had to be excluded because of changes of the routine blood values. No volunteer had a known history of immune disease, immunomodulating treatment, cancer, chronic terminal disease, severe cardiovascular disorder, substance abuse or severe trauma.

### Preparation of peripheral blood mononuclear cells (PBMC)

PBMCs were separated by standard density gradient centrifugation (Ficoll Paque; Biochrom AG, Berlin) for 20 min, 375x g, RT) from freshly drawn blood collected in lithium heparin-treated tubes (BD Vacutainer; BD Biosciences, San Jose, CA, USA), which was diluted 1:1 with phosphate-buffered saline (PBS). The cell ring was harvested and the cell suspension was washed twice in PBS. Cells were then suspended in FACS staining buffer and cell number was counted.

### Flow cytometry

Isolated PBMC were washed once and then incubated with fluorescently-labeled antibodies for 20 min at 4° C in staining buffer (PBS + 0.5% BSA). Antibodies used in this study included reagents specific for: CD3 (UCHT1) and CD56 (B159) from BD Pharmingen (San Diego, CA); VGF (D-20) and the detection antibody, donkey anti-goat IgG FITC, from Santa Cruz Biotechnology (Dallas, Texas, USA). Afterwards, data were collected on a FACS flow cytometer (Fortessa, BD Biosciences, Mountain View, CA) and analyzed using FACS DIVA software 6.1.3 (BD Biosciences, Mountain View, CA) and using FlowJo software (Treestar Inc., Ashland, OR). The data were analyzed using biexponential transformation function for complete data visualization.

### Stimulation of PBMC and CD3+ T cells in vitro

CD3+ T cells were isolated using Pan T cell isolation kit (Miltenyi Biotech, Bergisch Gladbach, Germany) and AutoMACS separation. PBMC and CD3+ T cells were resuspended in RPMI medium supplemented with 10% FCS and 1% antibiotics and plated at a density of 1×10^6^ cells/ml. PBMCs were stimulated with PMA/ Ionomycin (1mg/ml) and PHA (1mg/ml), CD3+ T cells with Dynabeads Human T-Activator CD3/CD28 (Lifetechnology Inc, Darmstadt, Germany) for 3 days.

For inhibition of VGF-mediated effects, anti-VGF (D20) or an isotype-matched control antibody was added to the cultures at day 0.

### Measurement of cell proliferation

To measure cell proliferation, PBMC and CD3+ T cells were stained with CSFE (5,6 Carboxyfluorescein Diacetate, Succinimidyl Ester; Molecular Probes, Lifetechnology Inc, Darmstadt, Germany) according to standard protocols. Cells were stimulated as described above. Cell proliferation was detected using flow cytometry.

### Measurement of cytokine production

After 3d stimulation, supernatants from PBMC and CD3+ T cells were harvested and stored at -20°C. The cytokine production was detected using a human Th1/Th2 Multiplex assay (affymetrix eBioscience, San Diego, CA, USA) according to the manufacturer's instructions.

### Treatment with rapamycin and cyclosporine A

Isolated PBMC were cultured for 24h in complete RPMI medium with or without the addition of rapamycin (200 ng/ml) or cyclosporine A (200 ng/ml). Afterwards, the cells were harvested and the expression of VGF on CD3+ T cells was detected using flow cytometry.

### Statistical analysis

Statistical analyses and regression analysis were performed with the SPSS 15.0 program (Statistical Product and Service Solutions, Chicago, IL, USA) and GraphPad Prism software. Pearson's correlation was used for analysis of correlation. For regression analysis, we used VGF+CD3+/ CD3+ as dependent factor and HB1Ac, BMI and age as independent variables. Difference statistic was calculated by univariate or one-way ANOVA. Statistical significance was defined as p<0.05.

## SUPPLEMENTARY TABLE


