# Supplementary material for: Age-related increase of VGF-expression in T lymphocytes
Source: Aging (Albany NY). 2014 May 4;6(6):440–53. doi: 10.18632/aging.100656 (PMC4100807; doi:10.18632/aging.100656)
Supplement: Supplementary file 1 [file aging-06-440-s001.pdf]

compartment requires monocytes and promotes inflammation in type 2 diabetes. J Immunol. 186:1162-1172.

67. Facchini A, Mariani E, Mariani AR, Papa S, Vitale M, Manzoli FA. Increased number of circulating Leu 11+ (CD 16) large granular lymphocytes and decreased NK activity during human ageing. Clin Exp Immunol. 1987; 68:340-347.

68. Vitale M, Zamai L, Neri LM, Galanzi A, Facchini A, Rana R, Cataldi A, Papa S. The impairment of natural killer function in the healthy aged is due to a postbinding defcient mechanism. Cell Immunol. 1992; 145:1-10.

69. Borrego F, Alonso C, Galiani M, Carracedo J, Ramirez R, Ostos B, Pena J, Solana R. NK phenotypic markers and IL2 response in NK cells from elderly people. Experimental gerontology. 1999; 34:253-265.

SUPPLEMENTARY FIGURES AND TABLES

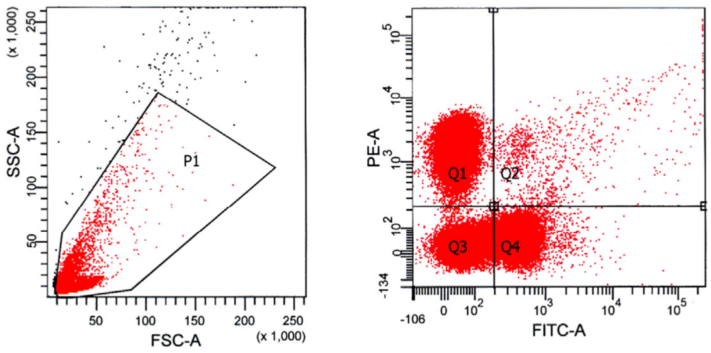

Supplementary Figure 1. Flow cytometry of VGF+CD3+ T cells. Isolated PBMC were stained with anti-VGF (D20) and anti-goat IgG-FITC and PE-labeled Abs against CD3 or CD56. First, the lymphocyte gate was set (left side) and then the frequency of VGF+ CD3+ cells detected (right side).

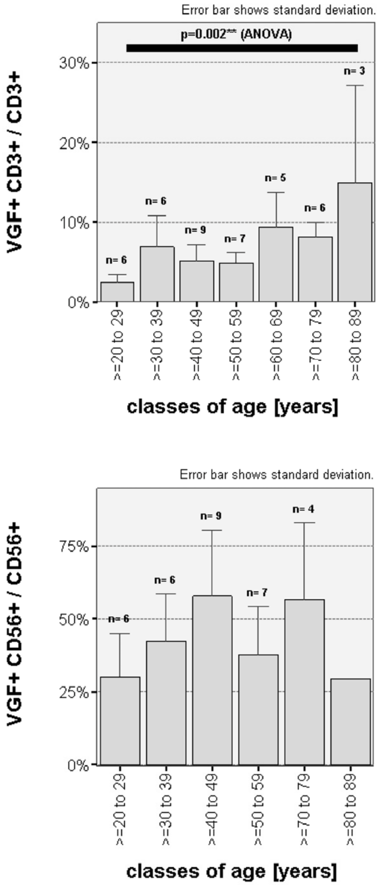

Supplementary Figure 2. VGF expression according to aged groups. The volunteers were grouped in ten-year-ranges. The relative numbers VGF+CD3+ T cells and VGF+CD56+ NK cells was calculated as percentage of the measured CD3 T cells or CD56+ NK cell numbers.

Supplementary Table 1. Demographic and blood parameters.

Routine blood parameters and demographic data, underlying diseases and medication as well as the number of VGF+CD3+ and VGF+CD56+ cells of the study cohort are shown in Suppl. Table 1.

	demographic data		routine blood parameters									
Case	age [years]	sex [f - female, m - male]	number of leukocytes [Gpt/l]	lymphocytes [%]	number of lymphocytes [Gpt/l]	PBMC [%]	number of PBMC [Gpt/l]	triglyceride [mmol/l]	choleresterol [mmol/l]	HDL [mmol/l]	LDL [mmol/l]	HbA1c [%]
**1**	22	m	7,0	0,50	3,50	0,56	3,92	0,81	5,05	1,53	3,15	5,1
**2**	22	f	10,1	0,31	3,13	0,36	3,64	1,09	3,46	1,98	0,98	5
**3**	28	f	10,0	0,42	4,20	0,47	4,70	0,80	4,39	1,54	2,49	5,1
**4**	28	m	7,0	0,30	2,10	0,35	2,45	1,02	3,54	1,26	1,82	4,5
**5**	25	f	10,1	0,41	4,14	0,46	4,65	1,28	5,15	2,53	2,04	4,9
**6**	32	f	7,5	0,35	2,63	0,38	2,85	0,92	6,25	2,42	3,41	5,5
**7**	42	f	8,0	0,27	2,16	0,32	2,56	0,93	4,52	4,51	2,59	5
**8**	25	m	7,5	0,26	1,95	0,32	2,40	1,04	4,30	1,21	2,62	4,9
**9**	38	f	11,2	0,25	2,80	0,30	3,36	0,80	5,00	1,27	3,37	5,3
**10**	38	m	9,8	0,47	4,61	0,59	5,78	2,72	4,41	1,13	2,04	5,5
**11**	35	f	4,3	0,27	1,16	0,34	1,46	0,40	4,04	1,49	2,37	5,7
**12**	58	f	4,6	0,40	1,84	0,48	2,21	0,85	4,13	1,41	2,33	5,7
**13**	48	f	4,5	0,32	1,44	0,38	1,71	1,16	4,75	1,59	2,63	5,3
**14**	52	m	7,8	0,27	2,11	0,34	2,65	0,90	4,76	1,24	3,11	5,8
**15**	42	m	5,4	0,21	1,13	0,28	1,51	2,25	5,45	1,34	3,09	5,4
**16**	52	f	6,7	0,20	1,34	0,24	1,61	1,13	5,32	1,79	3,02	5,3
**17**	32	m	8,4	0,46	3,86	0,51	4,28	0,69	4,68	1,34	3,30	5,3
**18**	58	m	6,0	0,40	2,40	0,47	2,82	0,62	5,94	1,37	4,29	5,8
**19**	55	f	4,8	0,49	2,35	0,53	2,54	0,55	6,69	2,37	4,07	5,7
**20**	55	m	7,6	0,30	2,28	0,35	2,66	1,19	4,25	1,38	2,33	5,3
**21**	45	m	8,3	0,19	1,58	0,26	2,16	2,49	5,40	1,34	2,93	5,7
**22**	55	m	4,7	0,24	1,13	0,31	1,46	2,50	5,18	1,65	2,39	5,9
**23**	45	f	6,6	0,36	2,38	0,40	2,64	10,46	6,83	1,26	-	5,2
**24**	35	m	4,8	0,35	1,68	0,41	1,97	0,85	4,98	1,29	3,30	5,3
**25**	42	f	6,5	0,25	1,63	0,29	1,89	1,49	5,56	1,47	3,41	5,6
**26**	45	m	4,6	0,35	1,61	0,42	1,93	2,86	6,12	1,22	3,60	5,2
**27**	40	m	7,4	0,37	2,74	0,49	3,63	2,27	6,41	1,08	4,30	5,7
**28**	46	f	9,4	0,36	3,38	0,04	0,39	0,64	5,84	1,85	3,70	5,5
**29**	64	f	5,4	0,31	1,67	0,36	1,94	1,33	5,66	2,29	2,77	5,6
**30**	63	m	4,8	4,00	19,20	0,48	2,30	3,22	6,43	1,40	3,57	5,9
**31**	88	f	5,7	0,34	1,94	0,43	2,45	1,41	7,20	1,91	4,65	5,7
**32**	75	f	6,9	0,18	1,24	0,27	1,86	6,96	9,73	1,16	-	6,3
**33**	67	f	7,4	0,27	2,00	0,37	2,74	2,11	6,12	1,94	3,22	6,1
**34**	79	m	6,9	0,18	1,24	0,25	1,73	1,82	3,48	1,04	1,61	5
**35**	64	f	5,7	0,36	2,05	0,54	3,08	1,38	4,65	1,61	2,41	5,8
**36**	82	m	5,1	0,34	1,73	0,49	2,50	0,75	3,75	1,18	2,20	6,7
**37**	74	f	7,5	0,15	1,13	0,22	1,65	0,90	6,95	2,39	4,15	5,8
**38**	86	m	5,8	0,32	1,86	0,40	2,32	0,86	3,95	1,77	1,79	5,1
**39**	72	m	5,4	0,20	1,08	0,26	1,40	1,05	5,08	1,79	2,81	5,2
**40**	76	f	6,4	0,16	1,02	0,23	1,47	0,56	7,01	2,21	4,55	5,6
**41**	73	f	9,0	0,23	2,07	0,29	2,61	1,72	6,38	1,89	3,71	5,9
**42**	61	f	5,7	0,21	1,20	0,26	1,48	1,01	5,95	1,56	3,93	5,6

demographic						disease			blood	
Case	age [years]	sex [f - female, m - male]	BMI [kg/m²]	body height [cm]	body weight [kg]	Mini Mental Status-Test	diseases	medication	VGF+ CD3+ / CD3+ [%]	VGF+CD56+/CD56+ [%]
**1**	22	m	n.a.	n.a.	n.a.	30			1,73%	5,30%
**2**	22	f	19,33	n.a.	n.a.	30			1,62%	20,78%
**3**	28	f	19,30	n.a.	n.a.	30			1,71%	33,90%
**4**	28	m	22,70	n.a.	n.a.	30			2,80%	46,15%
**5**	25	f	24,49	175	75	30			3,07%	41,18%
**6**	32	f	26,99	170	78	30			5,69%	35,63%
**7**	42	f	25,00	n.a.	n.a.	30			3,43%	64,29%
**8**	25	m	25,11	n.a.	n.a.	30			4,05%	33,33%
**9**	38	f	26,00	n.a.	n.a.	30			4,26%	60,00%
**10**	38	m	23,00	n.a.	n.a.	30			3,40%	46,15%
**11**	35	f	n.a.	n.a.	n.a.	30			10,87%	58,25%
**12**	58	f	n.a.	n.a.	n.a.	30			4,95%	38,71%
**13**	48	f	n.a.	n.a.	n.a.	30			6,91%	92,86%
**14**	52	m	23,84	175	73	30			7,34%	58,39%
**15**	42	m	32,14	185	110	30			6,06%	47,96%
**16**	52	f	n.a.	n.a.	n.a.	30			4,36%	56,34%
**17**	32	m	27,30	n.a.	n.a.	30			4,72%	16,67%
**18**	58	m	26,50	n.a.	n.a.	30			4,78%	42,03%
**19**	55	f	27,00	n.a.	n.a.	30			4,87%	21,29%
**20**	55	m	24,10	n.a.	n.a.	30			3,04%	14,24%
**21**	45	m	26,87	183	90	30			7,08%	56,90%
**22**	55	m	23,00	n.a.	n.a.	30	arterial hypertension	ramipril	5,04%	32,20%
**23**	45	f	27,10	163	72	30			3,95%	65,91%
**24**	35	m	29,32	180	95	30			12,61%	37,17%
**25**	42	f	n.a.	n.a.	n.a.	30			5,54%	41,67%
**26**	45	m	n.a.	n.a.	n.a.	30	arterial hypertension	metoprolol	1,87%	13,75%
**27**	40	m	29,61	181	97	30			8,03%	79,82%
**28**	46	f	29,40	174	89	30			2,95%	56,63%
**29**	64	f	n.a.	n.a.	n.a.	30			5,97%	-
**30**	63	m	n.a.	n.a.	n.a.	30			9,80%	-
**31**	88	f	n.a.	n.a.	n.a.	30	cadiac insufficiency, osteoporosis	phenprocoumon	27,85%	-
**32**	75	f	n.a.	n.a.	n.a.	30			7,69%	-
**33**	67	f	n.a.	n.a.	n.a.	30	arterial hypertension, hyperlipidemia, hyperuricemia	metoprolol, telmisartan, simvastatin, allopurinol	6,56%	-
**34**	79	m	n.a.	n.a.	n.a.	30	arterial hypertension, hyperlipidemia, chronic obstructive pulmonary disease, cardiac insufficiency	metoprolol, telmisartan, pentaerythrityltetranitrat, simvastatin, acetylsalicylic acid, spironolacton	10,73%	-
**35**	64	f	n.a.	n.a.	n.a.	30	arterial hypertension	enalapril	16,59%	-
**36**	82	m	n.a.	n.a.	n.a.	30	arterial hypertension, hyperlipidemia, hypothyroidism	metoprolol, ramipril, simvastatin, acetylsalicylic acid	13,32%	-
**37**	74	f	n.a.	n.a.	n.a.	29			9,89%	30,46%
**38**	86	m	n.a.	n.a.	n.a.	29	arterial hypertension	furosemid, metoprolol, acetylsalicylic acid	3,45%	29,34%
**39**	72	m	n.a.	n.a.	n.a.	30			5,84%	80,77%
**40**	76	f	26,90	159	68	29	arterial hypertension, hypothyroidism	ramipril, nebilolol, levothyroxin	7,46%	77,78%
**41**	73	f	n.a.	n.a.	n.a.	29	arterial hypertension, hyperlipidemia, hypothyroidism	enalapril, bisoprolol, acetylsalicylic acid	7,17%	37,09%
**42**	61	f	n.a.	n.a.	n.a.	29			7,98%	-

Supplementary Table 2. Correlation between blood parameters and age. The most important blood parameters and by FACS analysis received results were correlated with age and shown as Pearson correlation coefficient.

correlations

	age [years]		
	Pearson correlation coefficient	P-value(two-tailed)	N
number of leukocytes[Gpt/l]	-.396**	.009	42
lymphocytes [%]	-.388*	.011	42
number of lymphocytes[Gpt/l]	-.557**	.000	42
monocytes [%]	.218	.165	42
number of monocytes [Gpt/l]	-.093	.556	42
PBMC [%]	-.193	.221	42
number of PBMC [Gpt/l]	-.439**	.004	42
triglyceride [mmol/l]	.078	.622	42
choleresterol [mmol/l]	.335*	.030	42
HDL [mmol/l]	.026	.871	42
HbA1c [%]	.555**	.000	42
VGF+ CD3+ / CD3+ [%]	.536**	.000	42
VGF+ CD56+ / CD56+[%]	.204	.256	33

**. P-value < 0.005

*. P-Value <0.05
